# Pharmacological Strategies to Decrease Long-Term Prescription Opioid Use: A Systematic Review

**DOI:** 10.3390/jcm13247770

**Published:** 2024-12-19

**Authors:** Hannah Ellerbroek, Gerard A. Kalkman, Cornelis Kramers, Arnt F. A. Schellekens, Bart J. F. van den Bemt

**Affiliations:** 1 Department of Psychiatry, Radboud University Medical Center, 6525 GA Nijmegen, The Netherlands; arnt.schellekens@radboudumc.nl; 2 Nijmegen Institute for Scientist-Practitioners in Addiction (NISPA), 6525 HR Nijmegen, The Netherlands; 3 Department of Clinical Pharmacy, Canisius-Wilhelmina Hospital, 6532 SZ Nijmegen, The Netherlands; a.kalkman@cwz.nl (G.A.K.); kees.kramers@radboudumc.nl (C.K.); 4 Department of Pharmacy, Radboud University Medical Center, 6525 GA Nijmegen, The Netherlands; b.vandenbemt@maartenskliniek.nl; 5 Donders Institute for Brain, Cognition and Behaviour, Radboud University, 6525 EN Nijmegen, The Netherlands; 6 Department of Pharmacy, Sint Maartenskliniek, 6574 NA Nijmegen, The Netherlands

**Keywords:** prescription opioids, prescription opioid use disorder, prescription opioid misuse, opioid tapering, opioid rotation, opioid agonist treatment

## Abstract

**Background/Objectives**: As long-term prescription opioid use is associated with increased morbidity and mortality, timely dose reduction of prescription opioids should be considered. However, most research has been conducted on patients using heroin. Given the differences between prescription and illicit opioid use, the aim of this review was to provide an overview of pharmacological strategies to reduce prescription opioid use or improve clinical outcomes for people who experience long-term prescription opioid use, including those with opioid use disorder. **Methods**: We conducted a systematic database search of PubMed, Embase, CINAHL, and the Cochrane Library. Outcomes included dose reduction, treatment dropout, pain, addiction, and outcomes relating to quality of life (depression, functioning, quality of life). **Results**: We identified thirteen studies (eight randomized controlled trials and five observational studies). Pharmacological strategies were categorized into two categories: (1) deprescribing (tapering) opioids or (2) opioid agonist treatment (OAT) with long-acting opioids. Tapering strategies decreased opioid dosage and had mixed effects on pain and addiction. OAT with buprenorphine or methadone led to improvements in pain relief and quality of life, with a slight (non-significant) preference for methadone in terms of treatment retention (RR = 1.10 [CI: 0.89–1.37]) but not for other outcomes. Most studies had high dropout rates and a serious risk of bias. **Conclusions**: Tapering reduced prescription opioid doses had mixed effects on pain. OAT improved clinical outcomes without dose reduction. Based on our review findings, there is no clear preference for either tapering or OAT. Tapering may be considered first as it reduces dependency, tolerance, and side effects, but is associated with adverse events and not always feasible. OAT can be a suitable alternative. Non-pharmacological interventions may facilitate tapering. Further research is needed to identify novel pharmacological strategies to facilitate opioid tapering. **Registration:** PROSPERO 2022 CRD42022323468.

## 1. Introduction

Although opioids are effective short-term analgesics, there is no evidence to support their effectiveness in managing long-term pain [1]. In contrast, long-term use of opioids can lead to opioid-induced hyperalgesia [2] and is associated with a high incidence of adverse events, such as constipation, fatigue, dizziness, and drowsiness [3], as well as an increased risk of developing anxiety and depression [4]. Long-term opioid use can lead to tolerance (requiring increasing doses to sustain the initial effects), misuse, and prescription opioid use disorder (OUD) [5]. Patients using high doses of opioids (>50 mg oral morphine equivalence dose) have twice the odds of opioid-related mortality compared to those using lower doses [6]. Guidelines recommend considering tapering (decrementing opioid dose in set time intervals [7]) to a lower dose or discontinuation when opioid-related risks outweigh the benefits [8].

Tapering off long-term prescription opioid use can be challenging, as it induces withdrawal symptoms and may, at least temporarily, increase pain [9]. As a result, many patients continue to use opioids despite their desire to discontinue them [10]. Additionally, studies have shown that one-third to half of attempts to discontinue opioids are abrupt despite the associated risks [11,12]. Evidence on strategies for successful opioid dose reduction of prescription opioids is limited. Studies are mainly based on patients dependent on illicit opioids (see, for instance, [13,14] for meta-analyses). These patients often differ from those using prescription opioids in demographic characteristics, socioeconomic circumstances, support network, health care utilization, and chance of treatment completion [15,16].

Given the differences between prescription and illicit opioid use, the aim of this review is to provide a comprehensive overview of the effectiveness of pharmacological strategies to reduce opioid use or improve clinical outcomes for people who experience long-term prescription opioid use. Recent reviews have mainly focused on psychological support and non-pharmacological approaches [17,18]. A recent study by Wakeman et al. showed that opioid agonist treatment (OAT) with buprenorphine or methadone in patients with OUD (both illicit and prescribed opioids) was associated with reduced opioid-related morbidity compared to no treatment or psychological treatment, highlighting the importance of adequate pharmacological approaches [19]. A Cochrane review compared the effectiveness of buprenorphine and methadone OAT in patients dependent on prescription type, but not necessarily prescribed opioids [14]. The review found that OAT with buprenorphine and methadone was more effective in terms of treatment retention and reducing non-prescribed opioid use than non-opioid treatments, with a slight preference for methadone.

Both Wakeman et al. and the Cochrane review highlighted the potential of pharmacological strategies for patients with long-term prescription opioid use, including those with OUD [14,19]. However, an overview of the effectiveness of such strategies beyond OAT with buprenorphine or methadone is lacking. We, therefore, conducted a systematic review to synthesize an overview of the evidence for different pharmacological strategies to reduce prescribed opioid use or improve clinical outcomes.

## 2. Materials and Methods

We used a systematic review approach, conducted according to the Preferred Reporting Items for Systematic Reviews and Meta-Analyses (PRISMA) guidelines [20]. The review was registered in PROSPERO (CRD42022323468).

### 2.1. Data Sources and Searches

PubMed, Embase, CINAHL, and Cochrane Library databases were searched on 31 July 2024 for peer-reviewed, English-language studies without date restrictions. A search strategy for each database was developed with a research librarian. The search strings were crafted to include a variety of terms related to opioid-related disorders, treatment strategies, and pharmacological interventions while excluding irrelevant subjects such as pediatric, maternal, and non-opioid substance use studies. The detailed search strategy for each database is provided in Supplementary Materials S1. Additionally, backward citation searching (by checking the reference lists) and forward citation searching (identifying articles referring to an included paper using Web of Science) were applied.

### 2.2. Study Selection

Articles were included if they met the following criteria:

1. Focused on patients aged 18 or over with long-term use (>3 months) of prescribed opioids. We included all studies with patients with long-term prescription opioid use, irrespective of a diagnosis of prescription opioid use disorder (P-OUD) according to DSM-5 criteria [21] and irrespective of whether experiencing pain was an inclusion criterion;
2. Had one or more pharmacological interventional condition(s) aimed at reducing opioid use or improving functional outcomes. Studies with a psychological co-intervention were only included if both treatment arms were offered (but not necessarily received) the same co-intervention, allowing us to isolate the effects of the pharmacological intervention, and;
3. Were prospective studies.

Articles were excluded:

1. If they included participants requiring opioids for malignant or palliative pain;
2. If participants used illicit opioids as their most-used opioid. Illicit opioid use secondary to prescribed opioids was permitted;
3. If they were case reports.

Titles and abstracts were independently screened and selected for full-text assessment by different combinations of two reviewers (HE and AK or HE and BB). Disagreements were resolved by discussing with the third reviewer (AK or BB). Further screening for relevance and inclusion based on full-text articles was performed by different combinations of two reviewers (HE and AK or HE and BB). Any disagreements were discussed until a consensus was reached.

Although the PROSPERO registration specified the inclusion of only controlled trials, after registration, we decided to also include single-arm trials as well because of the limited number of controlled trials.

### 2.3. Data Extraction

Data extraction, according to an extraction format developed by the research team, was completed by one researcher (HE) and verified by another reviewer (AK or BB). Data extraction included bibliometric data, study characteristics, participant details, intervention details, and study baseline and outcome data. The primary outcome was opioid dose at follow-up, either of the baseline prescribed opioids or the rotation opioid if rotation to a long-acting opioid and subsequent tapering was chosen. Secondary outcomes were treatment dropout, pain, addiction outcomes (withdrawal symptoms and craving, opioid, and drug misuse), and outcomes relating to quality of life (depression, functioning, quality of life).

### 2.4. Risk of Bias Assessment

The risk of bias was assessed using the revised Cochrane Risk of Bias (RoB 2) tool for randomized trials [22] and the Cochrane Risk Of Bias In Non-randomized Studies—of Interventions (ROBINS-I) tool [23]. Two reviewers (HE, AK, or BB) independently assessed the risk of bias, and disagreements were resolved through discussion between the two researchers. The RCTs assessed with the RoB 2 tool were classified into one of three categories: low, some concerns, or high risk of bias. The observational studies assessed with the ROBINS-I tool were classified into one of four levels: low, moderate, serious, or critical risk of bias. A study was considered to be a high-quality study if it had a positive score (low risk for bias) on at least four out of five domains on the RoB 2 tool or six out of seven on the ROBINS-I tool. Risk of bias figures were created using the Risk of bias VISualization (robvis)-tool [24].

### 2.5. Data Synthesis and Analysis

The study flow was summarized according to PRISMA guidelines [20]. Each study was evaluated for (significant) differences in outcomes before and after the intervention and, if applicable, between interventions or between the intervention and a control condition. Due to the heterogeneity of the included studies, a quantitative method such as a meta-analysis (forest plots with risk ratios) could only be performed for treatment dropout since this was the only outcome that was consistently reported across more than one study (using Review Manager 5.4 [25]). Consequently, a descriptive best-evidence synthesis of the other predefined outcomes was performed.

## 3. Results

### 3.1. Search Results

Our search retrieved a total of 18,787 records from all databases combined. After removing duplicates, we screened 9491 unique articles for eligibility on titles and abstracts, leading to 258 articles eligible for full-text screening. During the full-text screening, we excluded 227 articles, mainly because the participants’ most-used opioid was illicit. From our initial search, we identified 30 articles covering 13 unique studies that met our inclusion criteria. Two articles covered one study [26,27] and 13 articles covered another study, the OPTIMA study [28,29,30,31,32,33,34,35,36,37,38,39,40].

**Figure 1:**
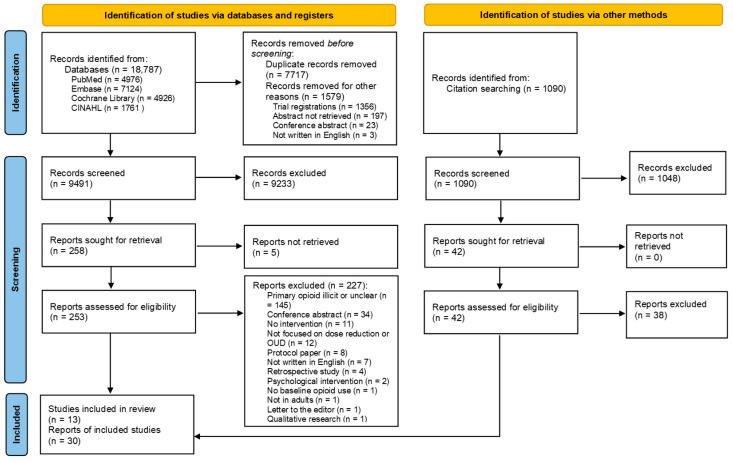
PRISMA flowchart.

After our initial screening, citation tracking yielded four additional articles published in the OPTIMA study [41,42,43,44]. In total, we identified 17 publications on the OPTIMA study [28,29,30,31,32,33,34,35,36,37,38,39,40,41,42,43,44]. When referring to the OPTIMA study in the results, we will refer only to the first article we identified in this study [28]. A description of the study flow and reasons for exclusion can be seen in the PRISMA flowchart (Figure 1).

### 3.2. Risk of Bias

Based on the risk of bias assessment, two studies were considered at some concerns on only one domain each and were therefore classified as high-quality [28,45]. Three studies were considered to be at critical risk of bias [46,47,48]. The remaining ten studies either had serious or a high risk of bias. The main sources of risk of bias were lack of blinding and missing data. The risk of bias rating for each individual study can be seen in Supplementary Materials S2.

### 3.3. Description and Narrative Synthesis of Studies

#### 3.3.1. Participants

The 13 studies included 806 participants, with a mean of 62 participants per study (range 8–270). Of the participants, 57% were male. All studies included participants on long-term opioid use, and eight out of thirteen studies only included participants who had a P-OUD [26,28,45,48,49,50,51,52]. Chronic non-cancer pain was an inclusion criterion in nine studies [26,46,47,49,51,52,53,54,55]. In the other studies, pain was not a requirement to participate.

#### 3.3.2. Types of Interventions

The interventions were categorized as follows:

1. Deprescribing interventions;
  a. Simple tapering without adjunctive medication;
  b. Tapering combined with adjunctive medication;
  c. Opioid rotation followed by tapering.
2. Opioid agonist treatment (long-term).

A short summary of interventions and findings can be seen in Table 1.

A more detailed summary can be found in Supplementary Materials S3. Below, we provide a narrative synthesis of each category.

#### 3.3.3. Category 1a—Simple Tapering Intervention

We identified one observational study by Nielsen and Jensen in which participants tapered their opioids without adjunctive medication [47]. The study included 22 participants with chronic non-cancer pain. The average baseline opioid dose was 86 mg Oral Morphine Equivalent (OME) dose. Participants had individualized tapering schedules, but specific tapering details (such as duration) or non-pharmacological support were not reported. On average, the participants reduced their opioid dose by 30%. Pain scores remained unchanged with the taper, but there was an improvement in depressive symptoms. However, 27% of participants dropped out due to worsening ailments, psychiatric symptoms, or an increase in addictive behavior. The risk of bias in this study was critical.

#### 3.3.4. Category 1b—Tapering with Adjunctive Medication

We identified four studies that tapered opioids while administering adjunctive medication. One study researched adding topical analgesics (diclofenac in 51 participants, ketoprofen and flurbiprofen each in 22, and non-specified-non-NSAID in 26 participants) [46], two studies assessed adding antidepressant medication (fluoxetine, doxepin) [48,55], and one study adding varenicline (vs placebo) [53]. All studies included participants using opioids in the long term, and one study included only those with P-OUD [48]. Three studies also had chronic pain as an inclusion criterion [46,53,55]. The studies on topical analgesics (*n* = 121, six months), fluoxetine (*n* = 8, two months) and doxepin (*n* = 35, six months) were all open-label studies [46,48,55]. The varenicline study (*n* = 21, three weeks) was a double-blind RCT [53].

One study (topical analgesics) did not report pre- and post-opioid doses [46], one study (fluoxetine) reduced opioid doses from an average of 33 mg to 6 mg OME [48] and two studies (varenicline, doxepin) tapered from around 100 mg OME to discontinuation [53,55]. During tapering, pain scores decreased for participants who tapered with varenicline (by 31.6%), as well as the placebo comparator arm in the same study (by 22.5%). Pain scores also reduced with topical analgesics (no sub-analysis per type of analgesics was conducted), while they remained stable for those who tapered with doxepin. The only study that reported on addiction outcomes was the study on varenicline vs. placebo. In this study, opioid withdrawal scores decreased over time in the tapering + varenicline condition, while it increased in the tapering + placebo condition [53]. Two studies (varenicline vs. placebo and fluoxetine) reported on depressive symptom severity, which improved in both studies in all conditions (with similar reductions in varenicline and placebo) [48,53].

The study on topical analgesics had a high dropout of 78%, mainly attributed to loss of follow-up. The other studies had a dropout between 0 and 43%, partly not explained by the authors and partly because “study participation distracted participants from other therapies” (varenicline study). The studies had some concerns (varenicline) [53], serious (doxepin) [55] or critical risk of bias (topical analgesics, fluoxetine) [46,48].

#### 3.3.5. Category 1c—Opioid Rotation Followed by Tapering

We identified four studies that rotated participants to another opioid and then tapered. Of these, three studies included participants with prescription opioid use disorder, while the fourth study included participants with long-term opioid use with a mean baseline dose of 280 mg OME [54]. Two RCTs (*n* = 12, six months/*n* = 113, 14 weeks) investigated tapering after rotation to buprenorphine/naloxone and compared tapering to stable maintenance dosing of buprenorphine/naloxone [45,49]. One RCT (*n* = 35, six months) rotated participants to sustained-release opioids (fentanyl, hydromorphone, morphine, oxycodone, tramadol, methadone; not defined how many participants received which opioid) and also compared tapering doses to stable maintenance doses [54]. In these RCTs, the stabilization period before tapering was one to four weeks. The fourth study, an RCT (*n* = 53, six weeks), rotated participants to either placebo, low- or high-dose extended-release tramadol. Tramadol was discontinued and replaced with placebo after one week for all participants [50].

One study (sustained-release opioids) reported only the initial opioid dose (280 mg OME) but not the dose at follow-up [54]. The other studies did not report the initial opioid dose but reported the post-intervention dose [45,49,50]. In the first buprenorphine taper vs. stable dosing study, there were no participants left in the taper condition at follow-up, while participants in the maintenance condition used an average of 9.8 mg buprenorphine [49]. In the second buprenorphine taper vs. stable dosing study, participants in the taper condition had tapered to discontinuation, while in the maintenance condition, they used an average of 15 mg buprenorphine [45]. In the tramadol study, participants had discontinued opioids in all conditions [50]. In the study that rotated participants to sustained-release opioids, pain scores remained unchanged after tapering [54]. The other studies did not report on pain. The addiction outcomes were mixed. The first buprenorphine study reported no change in concurrent illicit opioid use when tapering [49], while the other buprenorphine study reported increased use of illicit opioids and other drugs [45]. Two studies reported quality of life-related outcomes; one saw improvements in functioning with tapering buprenorphine [49], while the other saw a reduction in depressive symptoms when tapering with tramadol [50].

**Figure 2:**
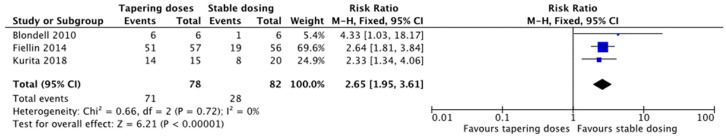
Risk for dropout with stable dosing compared to tapering doses after rotation [45,49,54]. Each ‘event’ is a dropout. The squares are the point estimate for each study, and square sizes corresponds to the weight they contribute to the pooled estimate. Rhomboid represents the pooled estimate.

In the three RCTs comparing tapering to stable dosing after rotation, dropout rates were significantly higher under tapering conditions (91%) compared to maintenance conditions (34%), RR 2.65 [CI: 1.95–3.61], Figure 2) [45,49,54]. Dropout was mostly due to the inability to reduce opioid doses; these participants switched to a maintenance dose. One study had some concerns (the second buprenorphine study) [45], the other studies were at high risk of bias [49,50,54].

#### 3.3.6. Category 2—Long-Acting Opioid Agonist Treatment

We identified five studies on opioid agonist treatment (OAT, substituting the currently prescribed opioid for a long-acting opioid). Among these studies were three RCTs (*n* = 54, six months/*n* = 19, six months/OPTIMA study, *n* = 270, 5.5 months) comparing buprenorphine/naloxone with methadone [28,51,52]. Furthermore, one observational study assessed OAT with buprenorphine/naloxone (*n* = 43, two months) [26]. The initial opioid dose was not reported in most studies. At follow-up, maintenance doses for buprenorphine ranged from 8 to 24 mg, and for methadone, from 29 to 120 mg.

Studies assessing pain, craving, depressive symptoms, and quality of life showed improvements both for buprenorphine and methadone OAT conditions. However, daily functioning results were mixed. One study (buprenorphine vs. methadone) found no significant difference pre- and post-intervention in both groups [51], while another study reported improvements in both buprenorphine and methadone conditions without a difference between the treatment arms [52]. The misuse of the prescribed or concurrently used illicit opioids and drugs decreased in all conditions over time for participants who were retained in treatment, but one RCT saw more illicit opioid use in the buprenorphine condition [51], while the OPTIMA study found more misuse in the methadone condition [28].

**Figure 3:**
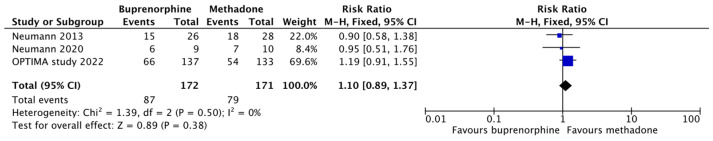
Risk for dropout after rotation to maintenance doses of buprenorphine and methadone [28,51,52]. Each ‘event’ is a dropout. The squares are the point estimate for each study, and square sizes corresponds to the weight they contribute to the pooled estimate. Rhomboid represents the pooled estimate.

Dropout rates in the three RCTs differed slightly between the buprenorphine (51%) and methadone conditions (46%) (RR of 1.10 [CI: 0.89–1.37], see Figure 3) [28,51,52]. Dropout was mainly due to a loss of follow-up, participants not picking up medication, and aberrant drug behavior. We cannot determine whether dose reduction was achieved because (1) the initial opioid dose was not reported in most studies and (2) there is no universally agreed formula to convert buprenorphine to oral morphine equivalents (or other opioids) [56]. The OPTIMA study had some concerns regarding the risk of bias [28], and the other two RCTs had a high risk of bias [51,52]. The observational study had a serious risk of bias [26].

### 3.4. Summary of Results

The interventions were divided into two main categories: deprescribing (tapering) and long-acting OAT. There were three types of deprescribing (tapering) studies: simple tapering without co-medication, tapering with adjunct medication, and rotation to another opioid followed by tapering. We identified one simple tapering study in which patients reduced opioid dose by 30%, but there was no change in pain or depressive symptoms. Four studies used adjunctive medication while tapering: topical analgesics; two types of antidepressants (fluoxetine, doxepin); and varenicline. Pain either remained stable or improved, and depressive symptoms improved. Four studies first rotated patients to another opioid (buprenorphine, sustained-released opioids, or tramadol) and then tapered. Results on pain and illicit opioid use were mixed while functioning generally improved. However, there was a very high drop-out in the tapering groups (compared to the stable dose comparator), up to 100%. For the OAT category, three studies compared buprenorphine/naloxone and methadone for long-term OAT. One study assessed OAT with buprenorphine/naloxone without a comparator. Both OAT with buprenorphine and methadone improved pain, cravings, and depressive symptoms, but drop-out was around 50%. There was no meaningful difference between buprenorphine and methadone in most outcomes and a slight preference for methadone regarding treatment retention. Overall, drop-out was high in nearly all studies included in this review, and the risk of bias was high.

## 4. Discussion

We systematically reviewed the literature on pharmacological strategies for people who have long-term prescription opioid use. These interventions aimed to reduce or discontinue opioid doses or to improve clinical outcomes such as pain and quality of life. Strategies were categorized as either (1) deprescribing/tapering (nine studies) or (2) long-acting opioid agonist treatment (OAT) strategies (four studies). We defined three subcategories of deprescribing: (1a) simple tapering interventions (1 study), (1b) tapering with adjunctive medication (four studies), and (1c) opioid rotation followed by tapering (four studies). Tapering successfully decreased opioid dosages with either no change or a reduction in pain scores and mixed effects on addiction outcomes. Studies on adjunctive antidepressants, nicotinic agonists, and topical analgesics showed some evidence of beneficial effects on pain. However, only one study directly compared adjunctive medication versus tapering only [53], limiting the strength of evidence.

Another approach was first rotating to long-acting or sustained-release opioids, followed by a taper. The control group in these studies consisted of participants who remained on a stable dose of the new opioid after rotation. Participants who tapered after rotation were more likely to discontinue treatment (91%) than those remaining on a stable dose after rotation (34%) (RR 2.65 [CI: 1.95–3.61]). Although these studies had higher dropout in the tapering conditions than those that tapered without rotation (category 1a and 1b), direct comparison is not possible as baseline conditions differ. Participants in studies that tapered after rotation study all had P-OUD or high-dose baseline opioid use (mean 280 mg OME), while the other tapering studies had lower baseline opioid use (between 33 and 107 mg OME). Furthermore, the period (one to four weeks) on the new opioid may have been insufficient to stabilize the new opioid before tapering [57]. The POATS study, a large study (*n* = 653) on rotation to buprenorphine-naloxone, followed by tapering, showed that a longer stabilization period (12 weeks) prior to tapering was associated with higher treatment retention compared to a two-week stabilization period. This study was not included in our review because it combined OAT and tapering with different intensities of counselling. However, it shows the benefits of a longer stabilization period. Interestingly, there was no difference in patients receiving counselling in the above outcomes [58]. Unfortunately, we did not find studies that directly compared tapering after rotation with tapering without first rotating, which would also be a relevant comparison.

In the studies on OAT, the participants’ current opioids were substituted with the long-acting opioids buprenorphine or methadone. Both medications were associated with improvements in pain, quality of life, and depressive symptoms. However, a subset of participants dropped out of treatment (in RCTs; 51% for buprenorphine and 46% for methadone, RR of 1.10 [CI: 0.89–1.37]). A recent systematic review (which included both participants with illicit and prescription OUD) also found a small but consistent preference for methadone in terms of treatment retention [59]. However, there was no preference for other domains (e.g., pain, addiction, quality of life). The choice of one over the other may be based on patient preference or patient-related contra-indications (mainly for methadone), clinician experience, availability, regulations, or cost [59,60]. Another consideration is the likelihood of future analgesic needs. Methadone may then be preferred, as additional opioids can be easily added in case of acute pain [61]. However, evidence is emerging that receptor blockade with the partial mu-opioid agonist buprenorphine can be overridden with high doses of full opioid agonists [62,63]. Another consideration for buprenorphine is the lower risk of respiratory depression, torsade de pointes, immunosuppression, serotonin toxicity, and hypogonadism than with methadone [64,65,66,67,68]. Furthermore, there is some evidence that buprenorphine can have a beneficial effect on mood and depression, due to its kappa opioid antagonism [69,70,71].

Tapering reduces the opioid dose, resulting in fewer negative effects such as dependency, tolerance, and side effects [72]. It can also reduce opioid-induced hyperalgesia [2]. However, tapering can be challenging for patients, as is reflected by the high dropout rates in the studies we included. Patients on long-term opioid therapy often experience psychological distress and low self-efficacy [73] and some patients report that their opioids help them cope with negative emotions [74]. Patients may also experience fear of worsening of pain or the onset of withdrawal symptoms [73,75,76]. Additionally, retrospective studies show that tapering prescription opioids is associated with an increased risk for adverse events, including overdose, start of illicit opioid use, and mental health crisis [12,77,78,79,80,81,82,83,84,85,86,87] (only one study showed a decrease in AEs with tapering [88]). Only two studies in our review assessed overdose as an outcome. They respectively reported an overdose in one patient (2.9% of their sample) [54] and three patients (1.1% of their sample) [30]. The other studies did not report on overdose, potentially due to their short follow-up periods ranging from a few weeks to a few months, while overdose may be more likely to happen later.

Consequently, it is crucial to provide psychological support while making a change in opioid therapy to help patients cope with distress and pain. Other systematic reviews in patients with chronic pain and long-term use of prescription opioids have shown that interdisciplinary pain programs, psychological support, behavioral management approaches, and group therapy can improve both tapering and OAT outcomes [18,89]. About half of the studies in our review included a psychological component, but the intensity was not always well described, making it difficult to determine its effects on treatment outcomes.

In addition, it is important to provide sufficient non-opioid pain relief during opioid tapering through non-opioid analgesia, physiotherapy, nerve blocks, or spinal cord stimulation [90,91]. Adjunctive medication, such as those studied in our review (antidepressants, nicotinic agonists), may also improve tapering outcomes, but data are limited. Furthermore, treatment of withdrawal symptoms with clonidine or lofexidine may also improve outcomes [92], and slower tapering may reduce dropout rates, as rapid treatment discontinuation has been associated with high drop-out rates, seeking opioids on the illicit market, and overdose [12,93]. In contrast, a slow taper (nine weeks or more) reduces the risk of emergency department visits or hospitalizations by two-thirds compared to a rapid taper [12]. The CDC guideline recommends a gradual tapering process, with a 10% reduction in dosage per month for patients who have been using opioids for over a year [94]. It is important to involve a patient in the decision to taper.

If a patient is unwilling to taper, is at risk of seeking opioids from the illicit market or other adverse events, or has previously failed tapering attempts, OAT can be a suitable alternative. OAT can prevent further dose escalation, stabilize patients, and is associated with reduced opioid-related harm in patients with illicit OUD [19]. OAT enables patients to focus on other aspects of recovery, such as psychological well-being and social- and vocational-functioning [72]. Improvements in these areas may lead to better-coping mechanisms and an enhanced quality of life. This, in turn, may create better circumstances to initiate tapering or opioid discontinuation at a later stage [58].

Identifying the most appropriate treatment for each patient can be challenging and should be a shared decision between patient and provider. Our review included three studies that analyzed baseline differences between treatment completers and non-completers. One of these studies found that baseline anxiety, depression, low general health, high opioid misuse, and fear of worsening pain were predictors for dropout (both in tapering and maintenance conditions) [54]. The OPTIMA study showed that low treatment satisfaction during follow-up predicted dropout [28,29]. Neumann et al. found no baseline differences between completers and non-completers [51]. Additionally, two OAT studies analyzed factors predicting treatment switching (switching from buprenorphine to methadone or vice versa). Women, participants with baseline fentanyl use, and those randomized to buprenorphine were significantly more likely to switch [40,51]. The authors concluded that the partial agonist effects of buprenorphine may not be sufficient to treat patients with very high opioid tolerance due to fentanyl use [40].

It is important to note that the objective of this review was focused on patients using long-term prescription opioids for chronic pain. We included both patients being prescribed opioids for pain and those prescribed opioids for a P-OUD (and often both combined). Though it may seem relevant to see these as two different patient populations, the development of OUD evolves over a continuum of gradually increasing use of opioids, development of opioid-related harm (tolerance and withdrawal, side-effects, etc.), and gradual loss of control [5,95], as in any other addiction. As a result, the distinction between those who have long-term excessive opioid use and opioid-related harm and those who have mild opioid use disorder (meeting only 2 DSM-5 criteria) is somewhat arbitrary [95]. From a pharmacological point of view, there is no reason to distinguish between these populations, as pharmacological approaches could be beneficial to both populations. Additionally, not all studies did, in fact, systematically assess whether or not OUD was actually present in their population (e.g., using structured clinical interviews). Therefore, we combined results for patients using prescription opioids with OUD diagnosis with results for those with long-term (and often high dose) opioid use without OUD diagnosis.

### 4.1. Future Research

Large-scale trials are needed to determine which participant factors are associated with the likelihood of success for each type of intervention. Also, future studies should include a wider variety of clinically relevant outcomes. Many studies only reported one or two outcomes, mostly treatment retention, dose reduction, and misuse of illicit opioids and drugs, while patients often consider outcomes such as pain and quality of life, including domains beyond health (e.g., relationships, psychological wellbeing) as more important [59,96,97]. Also, comparative studies are needed to identify whether tapering is easier with short- or long-acting opioids.

Beyond the pharmacological approaches studied here, psychedelics and cannabinoids may be an interesting add-on treatment to support opioid tapering [98,99]. Psychedelics have been associated with pain reduction [100,101] and lifetime use of psilocybin has been associated with a reduced likelihood of OUD (aOR: 0.70 [CI: 0.60–0.83]) [99]. Also, the co-administration of cannabinoids led to improvements in pain, sleep, and functioning [102,103] and opioid dose reductions ranging from 22 to 64% in patients with chronic pain [104,105].

Additionally, within OAT strategies, there is emerging data on micro-titration to buprenorphine. Unlike regular buprenorphine rotation, where patients go through a withdrawal period before switching to buprenorphine, micro-titration involves slowly adding buprenorphine with the current medication in small doses until it completely replaces the full opioid agonist at the receptor. This method minimizes withdrawal symptoms and may be associated with fewer dropouts. However, studies on micro-titration are limited to case reports and studies in mixed populations of both illicit and prescription OUDs [106]. Based on these data, micro-induction appears to be both safe and effective, but studies comparing micro-induction with conventional rotation in patients with pain on long-term opioid therapy are needed.

### 4.2. Limitations

This review has limitations. First, we excluded retrospective studies and case studies that may have provided additional evidence on different strategies for long-term opioid use. Second, we excluded studies that focused only on opioid rotation to improve pain without addressing dose reduction, treatment of P-OUD, or changes in functional clinical outcomes. These studies could have provided additional evidence of the analgesic effectiveness of different interventions.

## 5. Conclusions

We identified two treatment strategies for patients with long-term opioid use: tapering and OAT. Tapering strategies successfully decreased opioid dosages but had mixed effects on pain and addiction outcomes. A subset of tapering studies utilized adjunctive medication, showing some potential for beneficial effects on pain. The second strategy, OAT, resulted in improvements in pain and quality of life, with possibly a slight preference for methadone in terms of treatment retention. Based on our review, there is no clear preference for either tapering or OAT. However, tapering may be considered first over OAT as it reduces dependency, tolerance, and side effects, but patients can be at risk for adverse events. If tapering is unsuccessful or not feasible, OAT can be considered a viable alternative. Ultimately, the choice of treatment should be a shared decision between the patient and the provider. Any pharmacological intervention should be accompanied by non-opioid pain relief and psychological support where possible. Future research should (1) identify whether tapering is easier with short- or long-acting opioids, (2) explore novel pharmacological strategies such as psychedelics or cannabinoids as adjunctive medications, (3) explore the microtitration of buprenorphine for OAT, and (4) identify which intervention is most appropriate for which patient profile.

## Figures and Tables

**Table 1:** Summary of interventions and findings.

	Interventions	Duration	*n*	Population	Mean Dose at Follow-Up	Drop Out	Pain	Addiction	Quality of Life
1a. Tapering without adjunctive medication									
Nielsen 2022 [47]	Taper	3 w	22	Pain, 86 mg OME	60 mg OME	27%	=		+
1b. Tapering with adjunctive medication									
Gudin 2018 [46]	Taper + topical analgesic	6 m	121	Pain		78%	=		
Hooten 2015 [53]	Taper + varenicline	3 w	21	Pain, 98 mg OME	0 mg	30%	+	+	+
	Taper + placebo				0 mg	0%	+	−	+
Romach 2000 [48]	Taper + fluoxetine	2 m	8	P-OUD, 33 mg OME	6 mg OME	0%			+
Wang 2011 [55]	Taper + doxepin	6 m	35	Pain, 107 mg OME	0 mg	43%	+		
1c. Opioid rotation followed by tapering									
Blondell 2010 [49]	Bupr + taper	6 m	12	Pain, P-OUD	no pt left	100%		=	+
	Bupr + stable doses				9.8 mg bupr	17%		=	+
Fiellin 2014 [45]	Bupr + taper	14 w	113	P-OUD	0 mg	89%		−	
	Bupr + stable doses				15 mg bupr	34%		=	
Kurita 2018 [54]	SR opioids + taper	6 m	35	Pain, 280 mg OME		93%	=		
	SR opioids + stable doses					40%	−		
Lofwall 2013 [50]	Placebo + taper	6 m	53	P-OUD	0 mg	37%		+	+
	200 mg tram + taper				0 mg	29%		+	+
	600 mg tram + taper				0 mg	29%		+	+
2. Opioid agonist treatment (long-term)									
Neumann 2013 [51]	Bupr	6 m	54	Pain, P-OUD	14.9 mg bupr	58%	+	+	=
	Metd				29.1 mg metd	64%	+	+	=
Neumann 2020 [52]	Bupr	6 m	19	Pain, P-OUD	8–16 mg bupr	67%	+	+	+
	Metd				30–60 mg metd	70%	+	+	+
OPTIMA 2022 [28]	Bupr	5.5 m	270	P-OUD	max. 24 mg bupr	48%		+	+
	Metd				60–120 mg metd	41%		+	+
Schellekens 2021 [26]	Bupr	2 m	43	Pain, P-OUD, 328 mg OME	18.3 mg bupr	14%	+	+	+

w = weeks. m = months. OME = Oral Morphine Equivalent dose. Bupr = buprenorphine. pt = participant. SR = sustained-release. Metd = methadone. Tram = tramadol. Addiction = includes withdrawal, craving, misuse of opioids, and misuse of illicit drugs. Quality of life = includes quality of life, depression, functioning, ‘−’ = outcome worse at follow-up than at baseline. ‘=’ = outcome unchanged compared to baseline. ‘+’ = outcome improved at follow-up compared to baseline. An empty cell means that the respective study did not report the outcome.

## Data Availability

The data included in this manuscript are secondary data drawn from published literature. However, data extraction and risk of bias files are available from the corresponding author upon reasonable request.

## Supplementary Materials

The following supporting information can be downloaded at https://www.mdpi.com/article/10.3390/jcm13247770/s1; Supplementary Materials S1: database search strategy; Supplementary Materials S2: Risk of Bias assessment; Supplementary Materials S3: A detailed summary of results.
