# Supplementary material for: Pharmacological Strategies to Decrease Long-Term Prescription Opioid Use: A Systematic Review
Source: J Clin Med. 2024 Dec 19;13(24):7770. doi: 10.3390/jcm13247770 (PMC11728354; doi:10.3390/jcm13247770)
Supplement: Supplementary file 1 [file jcm-13-07770-s001.zip › Supplementary Materials S1.pdf]

<u>Additional file S1: search strings</u>

PubMed search string (July 31, 2024)

(("opioid related disorders"[MeSH Terms:noexp] OR "Morphine Dependence"[MeSH Terms] OR (("opiate"[Title/Abstract] OR "opioid*"[Title/Abstract] OR "morphin*"[Title/Abstract] OR "morfin*"[Title/Abstract] OR "oxycodone"[Title/Abstract] OR "Fentanyl"[Title/Abstract] OR "Hydromorphone"[Title/Abstract] OR "Tramadol"[Title/Abstract] OR "Codeine"[Title/Abstract] OR "Codeine"[MeSH Terms] OR "Morphine"[MeSH Terms] OR "Hydromorphone"[MeSH Terms] OR "Tramadol"[MeSH Terms] OR "Fentanyl"[MeSH Terms]) AND ("long term"[Title/Abstract] OR "prolonged"[Title/Abstract] OR "iatrogenic"[Title/Abstract] OR "misus*"[Title/Abstract] OR "escalat*"[Title/Abstract] OR "depend*"[Title/Abstract] OR "use disorder*"[Title/Abstract] OR "addict*"[Title/Abstract] OR "tolerance"[Title/Abstract] OR "Iatrogenic Disease"[MeSH Terms:noexp])))

AND ("opiate substitution treatment"[MeSH Terms] OR "opioid related disorders/drug therapy"[MeSH Major Topic] OR "opioid related disorders/rehabilitation"[MeSH Major Topic] OR "pharmaceutical treatment*"[Title/Abstract] OR "pharmacological treatment*"[Title/Abstract] OR "pharmaceutical therap*"[Title/Abstract] OR "pharmacological therap*"[Title/Abstract] OR "medication treatment*"[Title/Abstract] OR "medication assisted treatment*"[Title/Abstract] OR "opioid therap*"[Title/Abstract] OR "opiate therap*"[Title/Abstract] OR "strateg*"[Title/Abstract] OR "rotati*"[Title/Abstract] OR "substitut*"[Title/Abstract] OR "taper*"[Title/Abstract] OR "wean*"[Title/Abstract] OR "detox*"[Title/Abstract] OR "withdraw*"[Title/Abstract] OR "discontinuat*"[Title/Abstract] OR "cessation"[Title/Abstract] OR "convert*"[Title/Abstract] OR "Methadone"[Title/Abstract] OR "Buprenorphine"[Title/Abstract] OR "Ibogaine"[Title/Abstract] OR "suboxone"[Title/Abstract] OR "subutex"[Title/Abstract] OR "Naloxone"[Title/Abstract] OR "naltrexone"[Title/Abstract] OR "sustained release morphine"[Title/Abstract] OR "Methadone"[MeSH Terms:noexp] OR "Buprenorphine"[MeSH Terms] OR "Ibogaine"[MeSH Terms] OR "Naloxone"[MeSH Terms])

AND ("randomized controlled trial"[Publication Type] OR "controlled clinical trial"[Publication Type] OR "randomized"[Title/Abstract] OR "placebo"[Title/Abstract] OR "randomly"[Title/Abstract] OR "trial"[Title] OR "clinical trial"[Publication Type] OR "comparative study"[Publication Type] OR "cross over studies"[MeSH Terms] OR "random*"[Title/Abstract] OR "controll*"[Title/Abstract] OR "intervention study"[Title/Abstract] OR "experimental study"[Title/Abstract] OR "trials"[Title/Abstract] OR "before and after"[Title/Abstract] OR "interrupted time series"[Title/Abstract] OR "follow up studies"[MeSH Terms]))

NOT "pediatric*"[Title/Abstract]) NOT "pregnan*"[Title/Abstract]) NOT "matern*"[Title/Abstract]) NOT "neonatal"[Title/Abstract]) NOT "baby"[Title/Abstract]) NOT "newborn"[Title/Abstract]) NOT "children"[Title/Abstract]) NOT "infant*"[Title/Abstract]) NOT "heroin"[Title]) NOT "cocaine"[Title]) NOT "amphetamine"[Title]) NOT "methamphetamine"[Title]) NOT "alcohol"[Title]) NOT "marijuana"[Title]) NOT "cannabis"[Title]) NOT "nicotine"[Title]) NOT "smok*"[Title]) NOT "opium"[Title]) NOT ("animals"[MeSH Terms] NOT "humans"[MeSH Terms])) NOT "rat"[Title]) NOT "rats"[Title]) NOT "mouse"[Title]) NOT "mice"[Title]) NOT "rodent*"[Title]) NOT "dog"[Title]) NOT "dogs"[Title]) NOT "monkey*"[Title]) NOT "pigeon*"[Title]) NOT "comment"[Publication Type]) NOT "editorial"[Publication Type]) NOT "review"[Publication Type]) NOT "systematic review"[Title]) NOT "meta-analysis"[Publication Type]) NOT "meta-analysis"[Title]) NOT "case report"[Text Word]) NOT "consensus"[MeSH Terms]) NOT "guideline"[Publication Type]) NOT "retrospective study" [Title]) NOT “retrospective studies” [MeSH Terms]

Embase search string (July 31, 2024)

(Opiate addiction/OR morphine addiction/ OR ((opiate .ti,ab,kf. OR opioid* .ti,ab,kf. OR morphin* .ti,ab,kf. OR morfin* .ti,ab,kf. OR oxycodone .ti,ab,kf. OR fentanyl .ti,ab,kf. OR hydromorphone .ti,ab,kf. OR tramadol .ti,ab,kf. OR codeine .ti,ab,kf.) AND (long term .ti,ab,kf. OR prolonged .ti,ab,kf. OR iatrogenic .ti,ab,kf. OR misus* .ti,ab,kf. OR escalat* .ti,ab,kf. OR depend* .ti,ab,kf. OR "use disorder*".ti,ab,kf. OR addict* .ti,ab,kf. OR tolerance .ti,ab,kf.)))

AND (opiate substitution treatment/ OR opiate addiction/dt, rh OR pharmaceutical treatment* .ti,ab,kf. OR medication treatment* .ti,ab,kf. OR medication assisted treatment* .ti,ab,kf. OR opioid therap*.ti,ab,kf. OR strateg* .ti,ab,kf. OR rotati* .ti,ab,kf. OR substitut* .ti,ab,kf. OR taper* .ti,ab,kf. OR wean* .ti,ab,kf. OR detox* .ti,ab,kf. OR withdraw* .ti,ab,kf. OR discontinuat* .ti,ab,kf. OR cessation .ti,ab,kf. OR convert* .ti,ab,kf. OR methadone .ti,ab,kf. OR buprenorphine .ti,ab,kf. OR ibogaine .ti,ab,kf. OR suboxone .ti,ab,kf. OR subutex .ti,ab,kf. OR naloxone .ti,ab,kf. OR naltrexone .ti,ab,kf. OR sustained release morphine .ti,ab,kf.)

AND (exp controlled clinical trial/ OR randomized .ti,ab,kf. OR placebo .ti,ab,kf. OR clinical trial/ OR randomly .ti,ab,kf. OR trial.ti. OR comparative study/ OR crossover procedure/ OR random*.ti,ab,kf. OR controll*.ti,ab,kf. OR "intervention study" .ti,ab,kf. OR "experimental study" .ti,ab,kf. OR trials .ti,ab,kf. OR "interrupted time series".ti,ab,kf. OR follow up/)

NOT pediatric* .ti,ab,kf. NOT pregnan* .ti,ab,kf. NOT matern* .ti,ab,kf. NOT neonatal .ti,ab,kf. NOT baby .ti,ab,kf. NOT newborn .ti,ab,kf. NOT children .ti,ab,kf. NOT infant* .ti,ab,kf. NOT heroin.ti. NOT cocaine .ti. NOT amphetamine .ti. NOT methamphetamine .ti. NOT alcohol .ti. NOT marijuana .ti. NOT cannabis .ti. NOT nicotine .ti. NOT smok* .ti. NOT opium .ti. NOT (animal/ NOT human/) NOT rat .ti. NOT rats .ti. NOT mouse .ti. NOT mice .ti. NOT rodent* .ti. NOT dog .ti. NOT dogs .ti. NOT monkey* .ti. NOT pigeon* .ti. NOT editorial/ NOT exp review/ NOT systematic review .ti. NOT meta-analysis .ti. NOT case report/ NOT consensus/ NOT exp practice guideline/ NOT exp history/ NOT retrospective study/

Cochrane Library search string (July 31, 2024)

#1
([mh ^"opioid related disorders"] OR [mh "Morphine Dependence"] OR ((opiate:ti,ab,kw OR opioid*:ti,ab,kw OR morphin*:ti,ab,kw OR morfin*:ti,ab,kw OR oxycodone:ti,ab,kw OR fentanyl:ti,ab,kw OR hydromorphone:ti,ab,kw OR tramadol:ti,ab,kw OR codeine:ti,ab,kw OR [mh Codeine] OR [mh Morphine] OR [mh Hydromorphone] OR [mh Tramadol] OR [mh Fentanyl]) AND ("long term":ti,ab,kw OR prolonged:ti,ab,kw OR iatrogenic:ti,ab,kw OR misus*:ti,ab,kw OR tolerance:ti,ab,kw OR escalat*:ti,ab,kw OR depend*:ti,ab,kw OR ("use disorder*"):ti,ab,kw OR addict*:ti,ab,kw OR [mh ^"Iatrogenic Disease"])))

AND ([mh "opiate substitution treatment"] OR [mh "opioid-related disorders"/DT,RH] OR ("pharmaceutical treatment*"):ti,ab,kw OR ("pharmacological treatment*"):ti,ab,kw OR ("pharmaceutical therap*"):ti,ab,kw OR ("pharmacological therap*"):ti,ab,kw OR ("medication treatment*"):ti,ab,kw OR ("medication assisted treatment*"):ti,ab,kw OR ("opioid therap*"):ti,ab,kw OR ("opiate therap*"):ti,ab,kw OR strateg*:ti,ab,kw OR rotati*:ti,ab,kw OR substitut*:ti,ab,kw OR taper*:ti,ab,kw OR wean*:ti,ab,kw OR detox*:ti,ab,kw OR withdraw*:ti,ab,kw OR discontinuat*:ti,ab,kw OR cessation:ti,ab,kw OR convert*:ti,ab,kw OR methadone:ti,ab,kw OR buprenorphine:ti,ab,kw OR ibogaine:ti,ab,kw OR suboxone:ti,ab,kw OR subutex:ti,ab,kw OR naloxone:ti,ab,kw OR naltrexone:ti,ab,kw OR "sustained release morphine":ti,ab,kw OR [mh ^Methadone] OR [mh Buprenorphine] OR [mh Ibogaine] OR [mh Naloxone])

#2
#1 NOT pediatric*:ti,ab,kw NOT pregnan*:ti,ab,kw NOT matern*:ti,ab,kw NOT neonatal:ti,ab,kw NOT baby:ti,ab,kw NOT newborn:ti,ab,kw NOT children:ti,ab,kw NOT infant*:ti,ab,kw NOT heroin:ti NOT cocaine:ti NOT amphetamine:ti NOT methamphetamine:ti NOT alcohol:ti NOT marijuana:ti NOT cannabis:ti NOT nicotine:ti NOT smok*:ti NOT opium:ti NOT ([mh animals] NOT [mh humans]) NOT rat:ti NOT rats:ti NOT mouse:ti NOT mice:ti NOT rodent*:ti NOT dog:ti NOT dogs:ti NOT monkey*:ti NOT pigeon*:ti

in Trials

CINAHL Library search string (July 31, 2024)

(((TI opiate OR AB opiate) OR (TI opioid* OR AB opioid*) OR (TI morphin* OR AB morphin*) OR (TI morfin* OR AB morfin*) OR (TI oxycodone OR AB oxycodone) OR (TI fentanyl OR AB fentanyl) OR (TI hydromorphone OR AB hydromorphone) OR (TI tramadol OR AB tramadol) OR (TI codeine OR AB codeine) OR (MH Codeine+) OR (MH Morphine) OR (MH "Dihydromorphinone") OR (MH Tramadol) OR (MH Fentanyl)) AND ((TI "long term" OR AB "long term") OR (TI prolonged OR AB prolonged) OR (TI iatrogenic OR AB iatrogenic) OR (TI misus* OR AB misus*) OR (TI escalat* OR AB escalat*) OR (TI depend* OR AB depend*) OR (TI tolerance OR AB tolerance) OR (TI "use disorder*" OR AB "use disorder*") OR (TI addict* OR AB addict*)))

AND ((MH "Drug Substitution") OR (MH "Drug Tapering") OR (MH "Deprescribing") OR (TI "pharmaceutical treatment*" OR AB "pharmaceutical treatment*") OR (TI "pharmacological treatment*" OR AB "pharmacological treatment*") OR (TI "pharmaceutical therap*" OR AB "pharmaceutical therap*") OR (TI "pharmacological therap*" OR AB "pharmacological therap*") OR (TI "medication treatment*" OR AB "medication treatment*") OR (TI "medication assisted treatment*" OR AB "medication assisted treatment*") OR (TI "opioid therap*" OR AB "opioid therap*") OR (TI "opiate therap*" OR AB "opiate therap*") OR (TI strateg* OR AB strateg*) OR (TI rotati* OR AB rotati*) OR (TI substitut* OR AB substitut*) OR (TI taper* OR AB taper*) OR (TI wean* OR AB wean*) OR (TI detox* OR AB detox*) OR (TI withdraw* OR AB withdraw*) OR (TI discontinuat* OR AB discontinuat*) OR (TI cessation OR AB cessation) OR (TI convert* OR AB convert*) OR (TI methadone OR AB methadone) OR (TI buprenorphine OR AB buprenorphine) OR (TI ibogaine OR AB ibogaine) OR (TI suboxone OR AB suboxone) OR (TI subutex OR AB subutex) OR (TI naloxone OR AB naloxone) OR (TI naltrexone OR AB naltrexone) OR (TI "sustained release morphine" OR AB "sustained release morphine") OR (MH Methadone) OR (MH Buprenorphine) OR (MH Naloxone+))

AND ((PT "randomized controlled trial") OR (PT "controlled clinical trial") OR (TI randomized OR AB randomized) OR (TI placebo OR AB placebo) OR (TI randomly OR AB randomly) OR (TI trial) OR (PT "clinical trial") OR (PT "comparative study") OR (MH "Crossover Design") OR (TI random* OR AB random*) OR (TI controll* OR AB controll*) OR (TI "intervention study" OR AB "intervention study") OR (TI "experimental study" OR AB "experimental study") OR (TI trials OR AB trials) OR (TI "before and after" OR AB "before and after") OR (TI "interrupted time series" OR AB "interrupted time series"))
NOT (TI pediatric* OR AB pediatric*) NOT (TI pregnan* OR AB pregnan*) NOT (TI matern* OR AB matern*) NOT (TI neonatal OR AB neonatal) NOT (TI baby OR AB baby) NOT (TI newborn OR AB newborn) NOT (TI children OR AB children) NOT (TI infant* OR AB infant*) NOT (TI heroin) NOT (TI cocaine) NOT (TI amphetamine) NOT (TI methamphetamine) NOT (TI alcohol) NOT (TI marijuana) NOT (TI cannabis) NOT (TI nicotine) NOT (TI smok*) NOT (TI opium) NOT ((MH animals+) NOT (MH human)) NOT (TI rat) NOT (TI rats) NOT (TI mouse) NOT (TI mice) NOT (TI rodent*) NOT (TI dog) NOT (TI dogs) NOT (TI monkey*) NOT (TI pigeon*) NOT (PT comment) NOT (PT editorial) NOT (PT review) NOT (TI "systematic review") NOT (PT meta-analysis) NOT (TI meta-analysis) NOT "case report" NOT (MH consensus) NOT (PT guideline) NOT (TI retrospective study) NOT (MH "Retrospective Design")
