# Supplementary material for: Pharmacological Strategies to Decrease Long-Term Prescription Opioid Use: A Systematic Review
Source: J Clin Med. 2024 Dec 19;13(24):7770. doi: 10.3390/jcm13247770 (PMC11728354; doi:10.3390/jcm13247770)
Supplement: Supplementary file 1 [file jcm-13-07770-s001.zip › Supplementary Materials S2.pdf]

Additional file 2: risk of bias per study

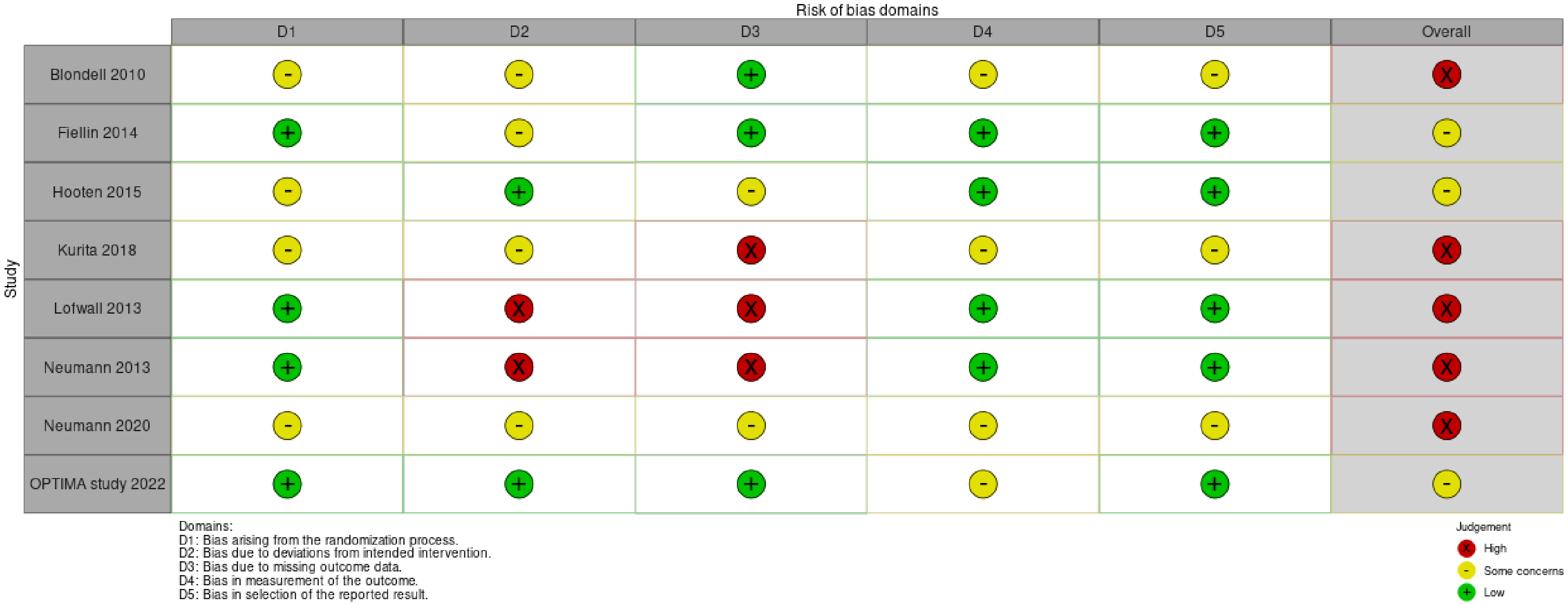

Figure S1: Risk of Bias for RCTs.

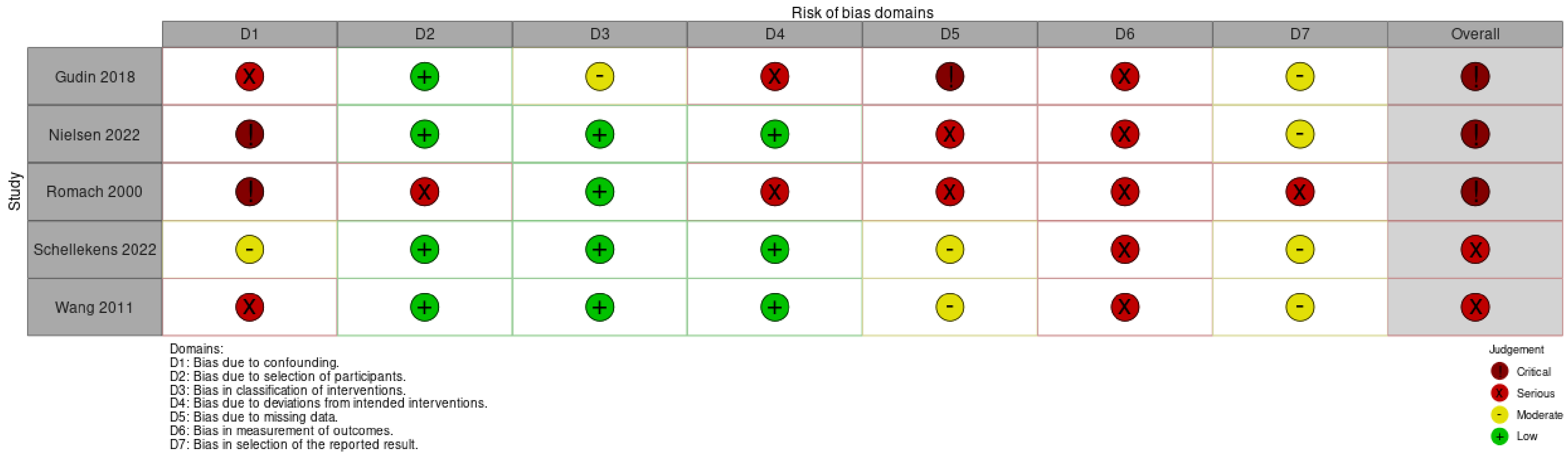

Figure S2: Risk of Bias for the non-randomized studies.
