# Supplementary material for: Pharmacological Strategies to Decrease Long-Term Prescription Opioid Use: A Systematic Review
Source: J Clin Med. 2024 Dec 19;13(24):7770. doi: 10.3390/jcm13247770 (PMC11728354; doi:10.3390/jcm13247770)
Supplement: Supplementary file 1 [file jcm-13-07770-s001.zip › Supplementary Materials S3.pdf]

Additional file S3: detailed summary of interventions and outcomes

	Design	Interventions	Concurrent therapies	Population	Dose at follow-up	% of dropouts and reason	Pain	Withdrawal and craving	Misuse of opioids or illicit drugs	Side effects	Quality of life related measures	Risk of Bias
1a. Tapering without adjunctive medication												
Nielsen, 2022 (Denmark)	Single arm, Follow up was taper duration + 3 weeks, open-label, outpatient.	Taper of current opioid (individualized schedule, timing not reported).	n.r.	n = 22<br>45% male<br>mean age: 59<br>CNCP + LTOT<br>mean 86 mg OME.	60 mg OME (30% dose reduction).	27%<br>*Psychiatric deterioration, addictive behaviour, worsening of illness.*	No effect of intervention.	n.r.	n.r.	n.r.	Depressive symptoms reduced.	Critical.
1b. Tapering with adjunctive medication												
Gudin, 2018 (U.S.)	Single arm, 6 months, open-label, unclear if in- or outpatient.	Start of topical analgesic (diclofenac in 42%, ketoprofen in 18%, flurbiprofen 18% or non-NSAID 22% of participants), while discontinuing opioids (titration or tapering details not provided).	n.r.	n = 121<br>45% male<br>mean age: 47<br>CNCP + LTOT<br>dose n.r.	78% drop-out<br>6% no change<br>4% dose reduced<br>12% discontinued opioids.	78%<br>*Lost to follow-up, loss of insurance, inadequate pain relief.*	Pain reduced.	n.r.	n.r.	Present in 1,7% of participants.	n.r.	Critical.
Hooten, 2015 (U.S.)	RCT (2 parallel arms)<br>3 weeks, double-blind, outpatient.	Taper of current opioid in 3 weeks combined with;<br>(1) varenicline (2 mg/day)<br>*or*<br>(2) placebo (2 mg/day).	Interdisciplinary pain treatment programme.	n = 21<br>72% male<br>median age: 47<br>CNCP + LTOT<br>mean 98 mg OME.	(1 and 2)<br>No opioid use (100% dose reduction).	(1) 30%<br>(2) 0%<br>*Distraction from other therapies.*	Pain reduced in both conditions.	Reduced withdrawal in varenicline condition and increased in placebo condition.	n.r.	No adverse events.	Depressive symptoms reduced in both conditions.	Some concern.
Romach, 2000 (Canada)	Single arm, 2 months, open-label, unclear if in- or outpatient.	Tapering of current opioid in 8 weeks combined with fluoxetine (20-40 mg/day).	Brief behavioural therapy.	n = 8<br>gender n.r.<br>mean age: 41<br>P-OUD<br>mean 33 mg OME.	6 mg OME (82% reduction).	0%	n.r.	n.r.	n.r.	n.r.	Depressive symptoms reduced.	Critical.
Wang, 2011 (Germany)	Single arm, 6 months, open-label, unclear if in- or outpatient.	Tapering current opioid (halving dose every 3 days) combined with daily doxepin (dose n.r.).	n.r.	n = 35<br>60% male<br>mean age: 49<br>CNCP + LTOT<br>mean 107 mg OME.	No opioid use (100% reduction).	43%<br>*Not explained.*	No effect of intervention.	n.r.	n.r.	n.r.	n.r.	Serious.

	Design	Interventions	Concurrent therapies	Population	Dose at follow-up	% of dropouts and reason	Pain	Withdrawal and craving	Misuse of opioids or illicit drugs	Side effects	Quality of life related measures	Risk of Bias
1c. Opioid rotation followed by tapering												
Blondell, 2010 (U.S.)	RCT (parallel arms), 6 months, open-label, Inpatient.	Rotation to **buprenorphine**/naloxone (1-2-day titration); one-month stable dose; followed by (1) tapering (rate not specified). *or* (2) continuation with stable dose.	12-step mutual help program, chemical dependency counselling.	n = 12<br>50% male<br>mean age: 45<br>CNCP + P-OUD<br>dose n.r.	(1) no data at follow-up.<br>(2) 9.8 mg buprenorphine.	(1) 100% *Changed to stable doses, aberrant drug behaviour.*<br>(2) 17% *High cost of buprenorphine.*	n.r.	n.r.	No change in illicit drug* use in both conditions compared to baseline.	n.r.	Functioning improved in both conditions.	High.
Fiellin, 2014 (U.S.)	RCT (parallel arms), 14 weeks, open-label, outpatient.	Rotation to **buprenorphine**/naloxone (12-day titration); one-month stable dose; followed by (1) tapering; reduction of 2 mg every 3 days. *or* (2) continuation with stable doses.	Counselling, psychoeducation.	n = 113<br>58% male<br>mean age: 30<br>P-OUD<br>dose n.r.	(1) no opioid use.<br>(2) 15 mg buprenorphine.	(1) 89% *Medication not picked up. Remained on stable doses.*<br>(2) 34% *Medication not picked up.*	n.r.	n.r.	More illicit opiate use in tapering condition (baseline n.r.).	n.r.	n.r.	Some concerns (High quality article).
Kurita, 2018 (Denmark)	RCT (parallel arms), 6 months, open-label, unclear if in- or outpatient.	Rotation to **methadone** or **sustained released opioids**; stable dose for 3 weeks; followed by (1) tapering; 10% reduction every 1-2 weeks. *or* (2) continuation with stable doses.	Access to psychologist/social worker/physiotherapist.	n = 35<br>40% male<br>mean age: 53<br>CNCP + LTOT<br>mean 280 mg OME.	(1 and 2) n.r.	(1) 93% *Could not reduce dose, changed treatment plan.*<br>(2) 40% *Changed treatment plan.*	Pain worsened in stable condition and remained unchanged tapering condition.	n.r.	n.r.	n.r.	n.r.	High.
Lofwall, 2013 (U.S.)	RCT (3 parallel arms), 6 weeks, double blind, inpatient.	Rotation to (1) **placebo** (2 weeks) *or* (2) extended-release **tramadol** 200 mg/daily (1 week); followed by placebo (1 week). *or* (3) extended-release **tramadol** 600 mg/daily (1 week); followed by placebo (1 week).	n.r.	n = 53<br>61% male<br>mean age: 30<br>P-OUD<br>dose n.r.	(1, 2 and 3) no opioid use.	(1) 37%<br>(2) 29%<br>(3) 29% *Withdrawal symptoms, wanted to use opioids, family problems.*	n.r.	Reduction of withdrawal scores for all conditions.	n.r.	More side effects in placebo condition than in tramadol conditions.	Depressive symptoms reduced in all conditions.	High.

Table S1: Summary of interventions and outcomes: tapering studies. CNCP: chronic non-cancer pain. LTOT = long-term opioid therapy (>3 months). POUD = prescription opioid use disorder. OME = oral morphine equivalent dose.

	Design	Interventions	Concurrent therapies	Population	Dose at follow-up	% of dropouts and reason	Pain	Withdrawal and craving	Misuse of opioids or illicit drugs	Side effects	Quality of life related measures	Risk of Bias
2. Opioid agonist treatment (long-term)												
Neumann, 2013 (U.S.)	RCT (2 parallel arms), 6 months, open-label, outpatient.	Rotation to (1) **buprenorphine**/naloxone (3-day titration); stable dosing. *or* (2) **methadone** (few weeks titration); stable dosing.	Chemical dependency treatment, Narcotics Anonymous).	n = 54<br>54% male<br>mean age: 38<br>CNCP + P-OUD<br>dose n.r.	(1) 14.9 mg buprenorphine.<br>(2) 29.1 mg methadone.	(1) 58%<br>(2) 64%<br>*Lost to follow up, did not comply, aberrant drug behaviour.*	Pain reduced in both conditions.	n.r.	Illicit drug* use reduced in both conditions, but more illicit drug use in buprenorphine condition.	Present in 65% of patients, no difference between conditions.	No difference in functioning before and after interventions.	High.
Neumann, 2020 (U.S.)	RCT (2 parallel arms), 6 months, open-label, outpatient.	Rotation to (1) **buprenorphine**/naloxone (3-day titration); stable dosing. *or* (2) **methadone** (few weeks titration); stable dosing.	n.r.	n = 19<br>32% male<br>mean age: 41<br>CNCP + P-OUD<br>dose n.r.	(1) 8-16 mg buprenorphine.<br>(2) 30-60 mg methadone.<br>*mean n.r.*	(1) 67%<br>(2) 70%<br>*Lost to follow-up, aberrant drug behaviour.*	Pain reduced in both conditions.	Craving reduced in both conditions.	Use of illicit opioids reduced for both conditions.	n.r.	Functioning improved and depressive symptoms reduced in both conditions.	High.
OPTIMA study 2022 (Canada)	RCT (2 parallel arms), 5,5 months, open-label, outpatient.	Rotation to (1) **buprenorphine**/naloxone stable doses. *or* (2) **methadone** stable doses.	n.r.	n = 270<br>65% male<br>mean age: 39<br>P-OUD<br>dose n.r.	(1) max. 24 mg buprenorphine.<br>(2) 60-120 mg methadone.<br>*mean n.r.*	(1) 48%<br>(2) 41%<br>*Lost to follow-up, did not pick up medication.*	n.r.	Craving reduced in both conditions.	More illicit opioid use in methadone condition (baseline n.r.).	(1) Adverse event in 6% of participants.<br>(2) Adverse event in 9% of participants.	Quality of life improved and depressive symptoms reduced in both conditions.	Some concerns (High quality article).
Schellekens, 2021; Veldman, 2022 (the Netherlands)	Single arm, 2 months, open-label, inpatient.	Rotation to **buprenorphine**/naloxone (7-day titration), stable dosing.	Morning meetings, creative activities.	n = 43<br>62% male<br>mean age: 47<br>CNCP + P-OUD<br>mean 328 mg OME.	18.3 mg buprenorphine.	14%<br>*Insufficient pain relief.*	Pain reduced.	Craving reduced.	Misuse behaviour of prescribed opioid reduced.	n.r.	Quality of life improved; depressive symptoms reduced.	Serious.

Table S2: Summary of interventions and outcomes: opioid agonist treatment studies. CNCP: chronic non-cancer pain. LTOT = long-term opioid therapy (>3 months). POUD = prescription opioid use disorder. OME = oral morphine equivalent dose.
